# Diagnosis of thoracic outlet syndrome with the lower trunk compression of brachial plexus by high-frequency ultrasonography

**DOI:** 10.1186/s12891-023-06762-7

**Published:** 2023-08-29

**Authors:** Dingzhang Chen, Wenqing Gong, Jing Wang, Jikun Hao, Rui Zhao, Minjuan Zheng

**Affiliations:** 1 Department of Ultrasound, Xijing Hospital, the Fourth Military Medical University, No. 127 Changle West Road, Xi’an, 710032 China; 2 Department of Hand-Surgery, Xijing Hospital, the Fourth Military Medical University, Xi’an, China

**Keywords:** High-frequency ultrasonography, Thoracic outlet syndrome, Brachial plexus

## Abstract

**Background:**

Thoracic outlet syndrome (TOS) with the lower trunk compression of brachial plexus (BP) is difficult to diagnosis. This study aimed to summarize the features of thoracic outlet syndrome (TOS) with the lower trunk compression of brachial plexus observed on high-frequency ultrasonography (HFUS).

**Methods:**

The ultrasound data of 27 patients who had TOS with the lower trunk compression of brachial plexus were collected and eventually confirmed by surgery. The imaging data were compared, and the pathogenesis of TOS was analyzed on the basis of surgical data.

**Results:**

TOS occurred predominantly in females (70.4%). Most cases had unilateral involvement (92.6%), mainly on the right side (66.7%). The HFUS features of TOS can be summarized as follows: (1) *Lower trunk compression.* HFUS revealed focal thinning that reflected compression at the level of the lower trunk; furthermore, the distal part of the nerve was thickened for edema (Affected side: 0.49 ± 0.12 cm vs. Healthy side: 0.38 ± 0.06, *P* = 0.009), and the cross-sectional area of brachial plexus cords was markedly greater on the injured side than on the healthy side (0.95 ± 0.08 cm² vs. 0.65 ± 0.11 cm², *P* = 0.004). (2) *Hyperechoic fibromuscular bands behind the compressed nerve* (mostly the scalenus minimus muscle). (3) *Abnormal bony structures*: cervical ribs or elongated transverse processes of the 7th cervical vertebra (C7). Surgical results showed that the etiological factors contributing to TOS were (1) muscle hypertrophy and/or fibrosis (100%) and (2) cervical ribs/elongated C7 transverse processes (20.7%).

**Conclusion:**

TOS with the lower trunk compression of brachial plexus can be diagnosed accurately and reliably by high-frequency ultrasound.

## Introduction

Thoracic outlet syndrome (TOS) is a group of diverse disorders involving compression of the nerves and/or blood vessels in the thoracic outlet region, including the subclavian artery, subclavian vein and trunks of the brachial plexus [1, 2]. TOS is subdivided into neurogenic, arterial, or venous TOS based on the compressed structure, in which neurogenic TOS accounts for approximately 90% of cases. Neurogenic TOS is mainly caused by the lower trunk compression of brachial plexus (the C8/T1 roots) [3]; patients may experience pain, numbness, paresthesia, and motor weakness [4]. The insidious onset and slow progression of symptoms usually result in delayed diagnosis.

At present, the diagnosis of TOS with the lower trunk compression of brachial plexus commonly relies on electromyography (EMG), CT and magnetic resonance imaging (MRI), in addition to medical history and clinical examinations. However, EMG cannot provide anatomical information regarding nerves and surrounding tissues, and the examination results are also affected by various factors [5]. Some authors state that CT scans can identify abnormalities in 30 to 60% of cases [6], but TOS caused by soft tissue is difficult to diagnose with CT. MRI is of value for the diagnosis of TOS: it identified a structure potentially responsible for TOS in 71% of the patients; was capable for hypertrophy of the anterior scalene muscle (81%) and for diagnosing a cervical rib (100% sensitivity and 100% specificity); but it is time consuming and costly [7]. An alternative is high-frequency ultrasonography (HFUS), a valuable technology that has long been applied for the assessment of superficial organs, tissues, and blood vessels, and it has also been applied recently to assess nerves [8, 9]. Previous studies on ultrasonic imaging of TOS are scarce and do not report complete surgical outcomes [10, 11]; furthermore, TOS is often missed or diagnosed as cervical spondylosis, inflammatory neuropathy and cubital tunnel syndrome. The aim of the present retrospective study was to summarize the ultrasound characteristics of TOS with the lower trunk compression of brachial plexus to provide references for TOS diagnosis.

## Methods

### Study subjects

From January 2011 to December 2021, 27 patients (29 sides, with 2 patients having bilateral lesions) presented with TOS involving the lower trunk compression of brachial plexus (19 females and 8 males). The patients were referred to the clinic because of hand and/or upper arm numbness and/or muscle atrophy. All patients underwent high-resolution ultrasound neurography, eighteen patients underwent EMG, and sixteen patients also underwent computed tomography (CT) scanning. The average age was 34.67 ± 14.20 years (13 to 68 years). All 27 participants were surgically confirmed to have TOS.

### Equipment and methods

A GE Logiq E11 ultrasound system with a 6–15 MHz linear array transducer (GE Medical Systems, Milwaukee, WI, USA) was used for HFUS. Ultrasonography was performed by two experienced sonographers (Dingzhang Chen, Minjuan Zheng). The subjects lay in a supine position and had both sides of the neck examined, and cross sectional imaging was also scanned for abduction of the arm in sitting position. Using the C7 vertebra (C7 is unique because it has a posterior tubercle only and can easily be found) as the positioning marker, the sonographers observed and recorded the continuity, size, morphology and echogenicity of the nerve roots (C5, C6, C7, C8, T1) and nerve trunks (upper, middle, and lower trunks). The subclavian artery (SCA) was identified, and the size of the brachial plexus in the supraclavicular area was evaluated. The morphology and echo characteristics of important anatomical structures, such as the subclavian artery, the transverse processes of the cervical vertebrae, and the muscles around the brachial plexus, were also observed.

### Statistical analysis

Statistical analyses were performed using statistical software (SPSS for Windows, version 21.0; SPSS, Chicago, IL, USA). Continuous variables are expressed as the mean ± standard deviation (SD), and categorical variables are expressed as percentages. The variables were compared using the t test or Fisher’s exact test. Probability (*p*) values < 0.05 were considered significant.

## Results

### Clinical features of TOS

TOS occurred predominantly in females (70.4%, 19/27). Most cases were located unilaterally (92.6%, 25/27), mainly on the right side (right: 66.7%, 18/27 vs. left: 33.3%, 9/27, *P* = 0.029). Most patients had upper limb numbness (79.3%) and muscle atrophy (75.9%) as symptoms. Upon clinical evaluation, TOS was often manifested as isolated finger weakness (75.9%), Froment’s sign (51.7%), a positive supraclavicular Tinel sign (41.4%), or a positive Adson (37.9%) or Wright (34.5%) test. EMG often showed neurogenic injury of the upper extremity involving 77.8% (14/18) of the lower trunk, and CT/X-ray diagnosis revealed that 31.3% (5/16) of the patients had an elongated C7 transverse process/cervical rib (Table 1).

**Table 1:** Clinical and ultrasound features of TOS (lesion location, 27 patients, n = 29 sides)

	n%, mean ± SD	P value
**Mean age (years)**	34.67 ± 14.20 (13~68)	
**Onset time (years)**	1.95 ± 1.85 (0.08~26)	
**Clinical manifestation, %(n)**		
Numbness	79.3%(23)	
Muscle atrophy	75.9%(22)	
Pain	27.6%(8)	
**Clinical evaluation**		
Isolated finger weakness	75.9%(22)	
Positive Froment’s sign	51.7%(15)	
Positive supraclavicular Tinel sign	41.4%(12)	
Positive Adson test	37.9%(11)	
Positive Wright test	34.5%(10)	
Positive Roos test	27.6%(8)	
**High-frequency ultrasonography**		
C5 root (diameter, cm)	0.31 ± 0.05	
C6 root (diameter, cm)	0.34 ± 0.05	
C7 root (diameter, cm)	0.37 ± 0.05	
C8 root (diameter, cm)	0.35 ± 0.05	
Lower trunk of brachial plexus (diameter, cm)		
Healthy side	0.38 ± 0.06	0.009
Affected side	0.49 ± 0.12	
Cross-sectional area of brachial plexus		
Healthy side (cm^2^)	0.65 ± 0.11	0.004
Affected side (cm^2^)	0.95 ± 0.08	
**Electromyography**		
Neurogenic injury of the upper extremity, involving the lower trunk of the brachial plexus	77.8%(14/18)	
**CT/X-ray**		
Cervical rib/elongated C7 transverse process	31.3% (5/16)	

### Ultrasonographic features of TOS

**Fig. 1:**
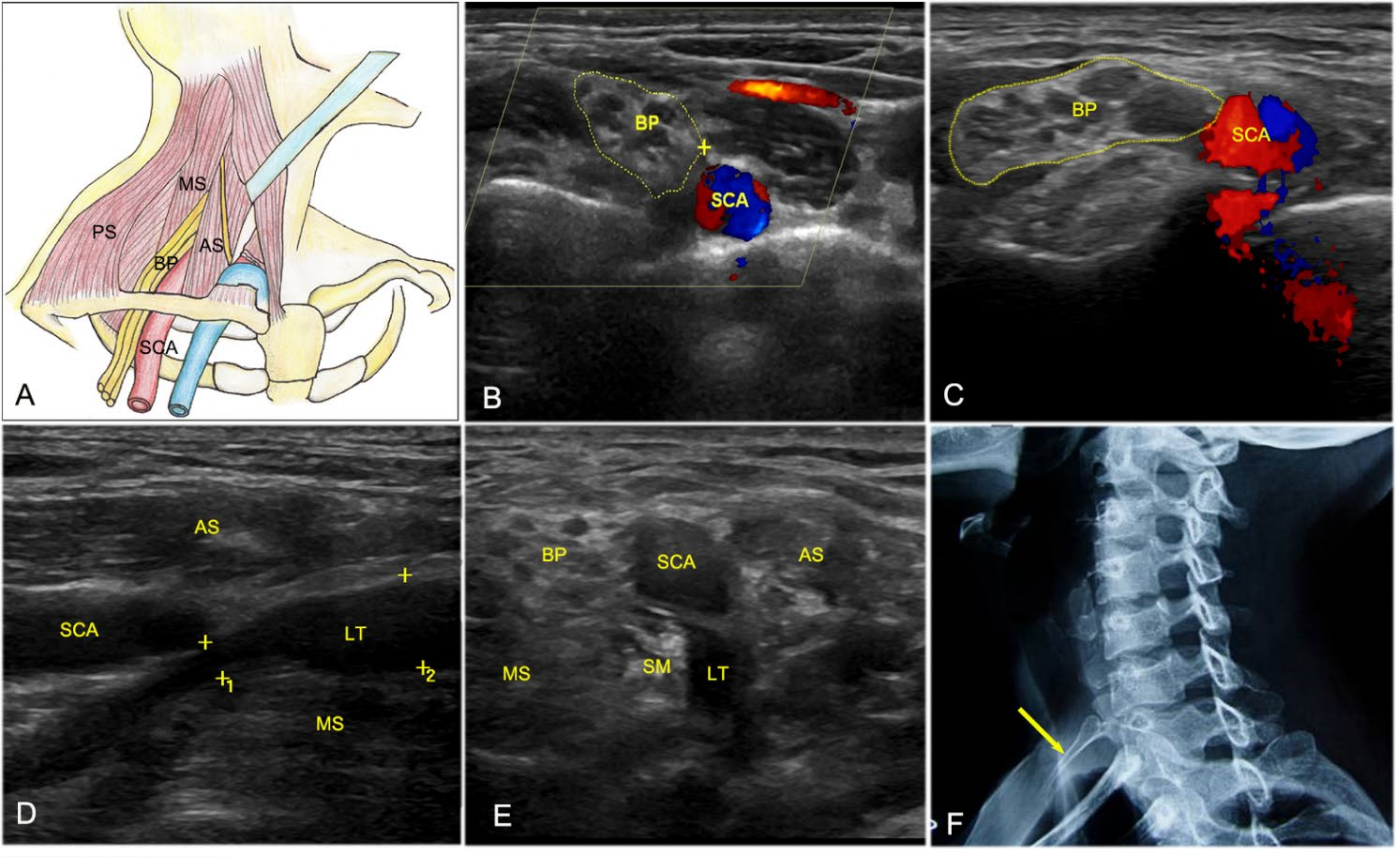
HFUS and CT images of TOS with the lower trunk compression of brachial plexus A. Anatomical diagram of the lower trunk of the brachial plexus B and C. A cross-sectional view showed that the area of the brachial plexus trunk was larger on the injured side (C) than on the normal side (B) D. Longitudinal view of the compressed LT and the compression caused by the MS. E. Short-axis view showing LT compression caused by the SM. F. X-ray image showing the cervical rib (arrow) BP, brachial plexus; LT, lower trunk, AS, anterior scalene muscle; SM, scalenus minimus muscle; MS, middle scalene muscle; SCA, subclavian artery

The HFUS imaging characteristics of TOS with a compressed lower trunk of the brachial plexus can be summarized as follows: (1) Lower trunk compression. HFUS showed focal thinning at the level of lower trunk due to compression. Additionally, the distal nerve was thickened for edema (Affected side: 0.49 ± 0.12 cm vs. Healthy side: 0.38 ± 0.06, *P* = 0.009, Fig. 1A and D), and the cross-sectional area of the brachial plexus cords was markedly greater on the injured side than on the healthy side (0.95 ± 0.08 cm² vs. 0.65 ± 0.11 cm², *P* = 0.004, Fig. 1B and C). (2) Hyperechoic fibromuscular bands (mostly in the scalenus minimus muscle) behind the compressed nerve **(**Fig. 1E**)**. (3) Abnormal bony structures: cervical ribs and elongated C7 transverse processes. HFUS showed hyperechoic bony structures with abnormal protrusions and shadows behind the deep surface of the compressed nerve root.

### Surgical results

**Fig. 2:**
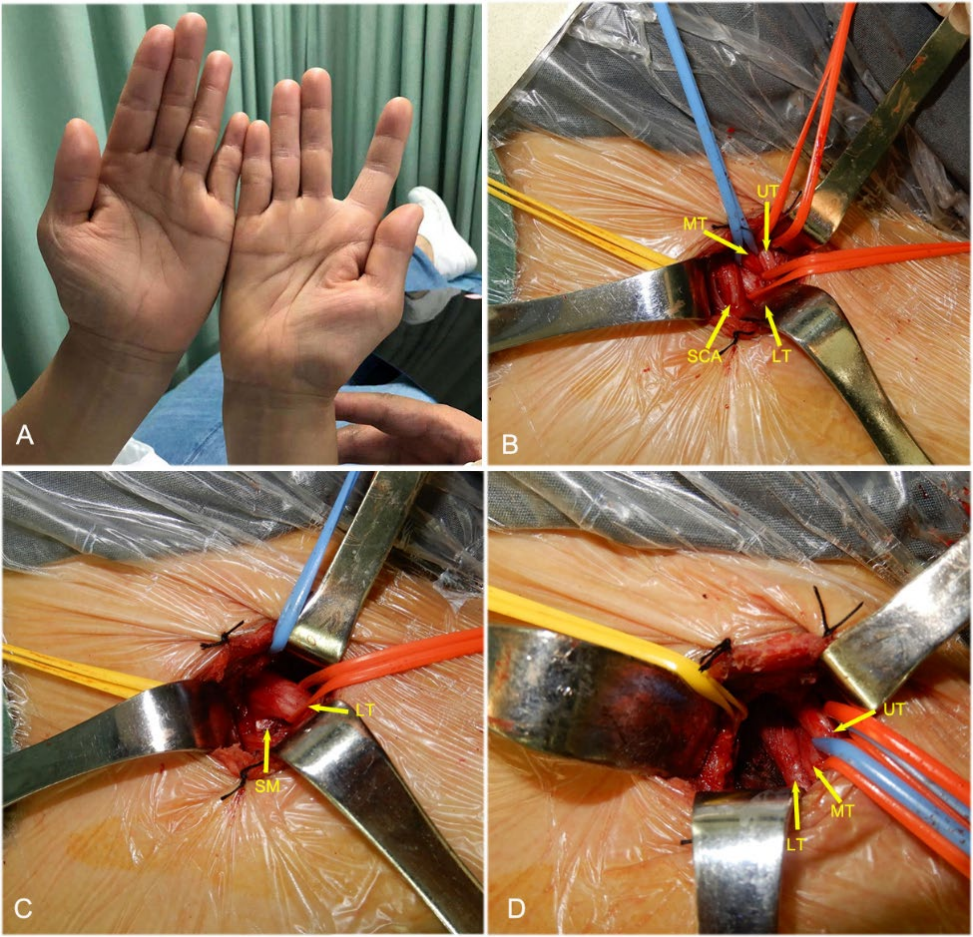
Intraoperative view of TOS with the lower trunk compression of brachial plexus A. Right thenar muscle atrophy B. Intraoperative image showing the brachial plexus (UT, MT, LT) and SCA. C. Intraoperative image showing hypertrophy and/or fibrosis (arrow) of the SM. D. Intraoperative image showing that the SM has been cut to release the LT. UT, upper trunk MT, middle trunk; LT, lower trunk; SM, scalenus minimus muscle; SCA, subclavian artery

Surgical results confirmed that the etiological factors of TOS were as follows: (1) Hypertrophy and/or fibrosis were present in the anterior and middle scalene muscles (23 sides, 79.3%), or hypertrophy, fibrosis, and/or bowstring-like changes were present in the scalenus minimus muscle (6 sides, 20.7%) **(**Figs. 1D and E and 2**)**. (2) Cervical ribs/elongated C7 transverse processes were present. In total, there were 6 cases of cervical ribs and 3 cases of elongated C7 transverse processes **(**Fig. 1F**)**.

## Discussion

TOS is difficult to diagnose, and the current approach to clinical diagnosis mainly relies on physical examination. Both the literature and our own experience demonstrate that HFUS is safe, inexpensive, readily available, and widely used in the evaluation of the perineural environment, fascicular echostructure, and nerve diameter, which are particularly valuable in the diagnosis and treatment of nerve tumors, compressive lesions, and nerve trauma [9, 10, 12–14]. However, there are few reports on TOS with compression of the brachial plexus in the existing literature. Therefore, we aimed to summarize the ultrasonographic features of TOS with the lower trunk compression of brachial plexus and preliminarily explore the diagnostic value of ultrasound in such diseases.

According to our data, TOS with a compressed lower trunk of the brachial plexus occurred predominantly in females (70.4%) and was usually unilateral (92.6%), with preferential involvement of the right arm (66.7%). This spatial distribution might be associated with the high prevalence of right-handedness, and risk factors might include repetitive movements and certain working positions, particularly in occupations requiring the use of the arms at an elevated angle (barbers, switchboard operators, assembly line workers, etc.) [6]. About 1–5% TOS patients accompanied subclavian artery stenosis in some severe cases [15, 16], but this phenomenon didn’t appear in our study.

HFUS plays an important role in the dynamic diagnosis of TOS and provides valuable information for clinical strategies. For patients with TOS involving the lower trunk compression of brachial plexus, successful surgery requires a thorough search for abnormal nerve morphology and any lesions in the perineural environment that are responsible for compression; preoperative positioning is also crucial [17]. In this study, the observed abnormalities in nerve morphology included focal thinning at the point of lower trunk compression and prominent thickening of the nerve (0.49 ± 0.12 cm) distal to the compression site, with an increase in the cross-sectional area of the nerve cords (affected side: 0.95 ± 0.08 cm²; healthy side: 0.65 ± 0.11 cm², *P* = 0.004). The perineural environment contained hyperechoic fibromuscular bands (mostly in the scalenus minimus muscle) behind the compressed nerve, and abnormal bony structures (cervical ribs and elongated C7 transverse processes) were also observed. However, hypertrophy or fibrosis of the scalenus minimus muscle could be found only in some patients, and this muscle was difficult to distinguish from the middle scalene muscle by HFUS. Arányi et al. [10]. identified a hyperechoic fibromuscular structure at the medial edge of the middle scalene muscle, which indented the lower trunk of the brachial plexus (‘‘wedge-sickle sign’’), would consistent with the structure of the scalene minimus muscle. C7 has only a posterior tubercle, makes the C7 transverse processes easy to identify [8]. If abnormal bony structures are seen along the deep surface of the nerve root, they should be noted.

On the basis of the surgical results, we found that the main etiological factor of TOS was muscle hypertrophy and/or fibrosis, with hypertrophy and/or fibrosis of the anterior or middle scalene muscle accounting for 79.3% of cases. We have observed that most TOS patients have relatively long necks [18]. The anterior and middle scalene muscles are prone to fibrosis when stretched for a long time; the fibrotic muscles, like a pair of scissors, would clamp the brachial plexus and especially the lower trunk where it passes between them. Preoperative HFUS showed that the cross-sectional area of the brachial plexus was greater on the lesion side than on the healthy side, and the lower trunk nerve was locally thickened, confirming the existence of lower trunk compression. Compression is often relieved surgically by cutting the anterior scalene muscle. Another important etiology of TOS is hypertrophy and/or fibrosis of the scalenus minimus muscle, which is a separate fibrous band, often arises from seventh and/or sixth cervical vertebrae and insert to the first rib, and occurs in 71.7% of TOS patients [19]. This population often combine with cervical ribs or elongated C7 transverse processes, which change the shape of the scalenus minimus muscle (in our patients, mostly behind the subclavian artery and the lower trunk of the brachial plexus), elevate the lower trunk, and cause the nerve to be compressed against the anterior scalene muscle. This type of compression is often relieved surgically by cutting the anterior scalene muscle and scalenus minimus muscle.

### Limitations

There are limitations to this study. For example, the total sample size was small, at 27 subjects. In addition, some patients did not undergo CT examination, and electromyography was performed in only 18 cases. Furthermore, postoperative follow-up data were not collected.

## Conclusion

HFUS is valuable for TOS diagnosis because it reflects both the morphology and the location of brachial plexus lesions and can detect muscular or bony factors causing nerve compression, providing important information for clinical treatment and postoperative follow-up.

## Data Availability

Data are available upon reasonable request via open or restricted access through a strict controlled access procedure request to the corresponding author.

## Abbreviations

TOS: Thoracic outlet syndrome
BP: brachial plexus
HFUS: high-frequency ultrasonography
AS: anterior scalene muscle
SM: scalenus minimus muscle
MS: middle scalene muscle
SCA: subclavian artery
MT: middle trunk
LT: lower trunk
CT: computed tomography
SD: standard deviation
